# Extracts from Lectures on Operative Dental Surgery

**Published:** 1887-07

**Authors:** William St. George Elliott


ARTICLE VII.

### EXTRACTS FROM LECTURES ON OPERATIVE DENTAL SURGERY.

BY WILLIAM ST. GEORGE ELLIOTT, M. D., D. D. S.

[Delivered at the National Dental College.]

Gold as a filling material has been used for a long time, but there is no evidence that it was used by the ancients; it has been stated that gold stopping have been found in some of the Egyptian mummies, but more thorough examination has shown that what was taken for stoppings was the ornamental gilding of the lips, more or less common at that early period. We cannot go back, I think, beyond the latter half of the last century. It was mentioned by Fauchard, 1785, and was in common use about 1800. The first American record we have is of some work done in London by a Mr. Waite about 1815, although it is quite possible that Woofendale first introduced it into America about 1795.

Gold, as used for stopping teeth, is not the commercial article classed as pure, and worth some £4 per ounce, but as made by different manufacturers, is not only of great purity, but of marked ductility.

Anyone who has done much in alloying gold for dentures, solder, &c., knows how easily this ductility is removed; a mere fraction of 1 per cent. of arsenic, or lead, is quite destructive to this necessary characteristic—indeed the copper and silver ordinarily used to alloy with, must be of great purity, or at least, must contain as little as possible of the objectionable materials. We require in gold great purity, not as some seem to think that it may be cohesive, nor on account of the ductility, as gold may be alloyed largely with some metals without materially injuring this property or destroying its cohesive character, but to withstand the secretions of the mouth; the purest gold will discolour in some months, not necessarily in those baldly cared for, as it sometimes noticed where the greatest care is taken, but in those mouths where there seems to be a development of *aqua regia*, as I have invariably noticed that when discolouration does exist, it is apparently the purple of Cassius (a double salt of gold and tin).

Gold, as used in the arts, is not only comparatively impure, as it contains more or less alloy, but it is beaten out much thinner than we use it. 200,000 sheets are required to make an inch in thickness.

While practising in Japan, I was in the habit of procuring some gold from China, made by the natives; it was apparently pure, and as ductile as that furnished by the depots, but I could not get it thinner than No. 60 or 80. As thick foil, it seemed to answer every requirement.

The Portuguese coins called Johanns, on account of their purity, were formerly used by the profession; at that time, early in the century, every dentist made his own gold, by rolling down these coins to a thickness of No. 10 or 20, and it was not until 1835, as far as I can ascertain, that the manufacture of gold for dental purposes was regularly taken up, Abbey, of Philadelphia, having commenced at that time.

In 1840, Dr. Westcott, of New York, called attention to a peculiar property of gold foil, it sometimes becoming sticky and thus objectionable. This property was subsequently utilized by Dr. Arthur, of Baltimore, in 1855. Before this, however, in 1853, Dr. Watt, of New York, and Mr. Joseph Barling, of Maidstone, Kent, brought out about the same time a new form of gold. It was then, and still is, called sponge gold, as well as crystal gold, &c.; it is made by precipitating from a solution of the chloride of gold the pure metal, by the assistance of electricity. It resembles a piece of exceedingly fine sponge, and is usually prepared for the cavity by tearing portions from the mass of the necessary size, annealing them, and forcing them into position, either with serrated hand instruments or mallet pluggers. I have long since abandoned the primitive mode of preparing it referred to, instead of which I use a rotary knife of one inch in diameter, and driven by the dental engine; it is best used at a high speed, 3,000 revolutions per minute, and will cut the gold into any sized blocks required without the least condensing it.

After many years' use of foil, I gradually took up sponge gold, and now use it exclusively. No. 1 and 2 of Watts' I find best suited to my requirements; the advantages I think, it possesses over foil are: it is more plastic, and lends itself more fully to the necessary packing against the walls of the cavity; unlike foil, it does not ball up under the plugger, nor block the way to undercuts, &c. It is more readily attached to the walls of the tooth, and is consequently not so easily displaced; it requires less retaining points and undercuts, it produces a hard surface by malleting, and retains a burnish much longer than foil. As a possible objection, I think it may not be quite as strong as foil for bridging from one point to another.

Soft foil, or that form of gold that came first into use, was employed as a filling material, just as paper would be used for the same purpose; the general principle recognized being that each piece or pellet should go from the bottom to the top of the cavity, like, in fact, cigars in a case.

As originally used, the small pellets or sections of rope were forced by hand pressure against the walls of the cavity, other pieces were added and packed laterally, until the cavity was fairly well filled; then a pointed or wedge-shaped instrument was forced into the mass, and the hole enlarged by lateral motion. Other pieces were added, as far as possible, until no more could be introduced; the projecting mass was now condensed by plugger and burnisher, and finished off.

Subsequently, foil, folded in the form of tape, was used in the same way, while, still later, this tape was wound round a bit of wire or five-sided broach into a cylinder.

These were made of uniform length for each cavity, but varied in thickness. Cylinders were generally used with a foot-shaped instrument, and were condensed from the distal end of the cavity forward.

Since the introduction of the mallet about 1868, they are frequently consolidated by this means, and when the cavity is completely filled, they are malleted on the surface.

The cylinders, as originally made by hand, were small and hard, being wound tightly around the wire.

They are now usually made light and soft, as by many operators they are also used, after annealing, in cohesive work. The use of the hard cylinders has been largely discontinued, although they have some advantages over the others.

It requires more skill to use non cohesive foil than the cohesive, and as there is some elasticity in a stopping made of this material, it preserves the tooth better; that is, a soft foil operator will be more successful in the class of cavities he will undertake, than one who only works with cohesive gold.

The class of cavities suitable for this gold must have four walls, more or less, while, of course, cohesive gold can be put almost anywhere that anchorage can be secured.

I hope, gentlemen, you will all be eclectic in your practice; use soft foil when you can do so, for generally you can make a successful operation in half the time you can with cohesive.

Of course, you will aim to become expert operators with both kinds, and you must also not forget the virtues of amalgam and oxyphosphates.

To return again to cohesive foil. Dr. Jack, by the use of retaining pits, was enabled to overcome some of the difficulties first experienced in the use of this material, and it rapidly became popular, introducing the era of contour fillings (which have done much to advance our profession, but which, like other good things, may have been carried too far,) I should think that not half the cases are worth the labour, pain, and money spent on them, particularly now that we have something so much better in gold crowns.

The union of surfaces in cohesive foil, although sufficient is at best but partial, at least as shown in the plug. We often hear of stoppings being taken out of a tooth or experimental matrix, and rolled out into a sheet as proof of their solidity (it is the rolling out which solidifies them); the force applied in the mouth is not sufficient, nor are the instruments adapted to such a result. They should have ovoid smooth points ordinarily like the goldbeater's hammer face, and the force applied at right angles to the surface of the filling; consequently such instruments and the force necessary, are inadmissable in the mouth.

We have to use serrations to prevent our pluggers from slipping, and the serrations do not leave the gold in the best condition for the union of surfaces.

I am in the habit of using occasionally for flat work, like in erosions, a flat square-edged, smooth instrument, and where the cavity is accessible and square with the instrument, one with an ovoid face, because it is only with a surface of this kind that you can expect to spread the gold and thoroughly condense it.

Now while it is not necessary to get perfect union between the pieces of gold in order to save teeth and retain a fair surface, do not for a moment undervalue condensation; nothing will condemn your work as much as lack of solidity, no feature is as important. A thoroughly solidified plug is retained much more firmly by the tooth, and will require more force to dislodge it, less undercuts are necessary, there is less give, to produce loosening; in every respect, for the work, if not for the patient's feelings, solidity is a most desirable quality. Do not, gentlemen, fall into the error which led me astray for some years. Do not think that surface malleting will take the place of homogeneous solidity, I first learned this fact by noticing in old fillings that those portions which had a hard foundation, as the edge of the enamel, &c., kept the best surface. Your stopping must be solid at the start, or it will loosen, and must be solid all through. Now, to get this desirable quality, time is necessary. Fine gold cohesive work generally means slow work; for this reason, the mechanical mal let is so valuable, and next to it I place self malleting. One can strike for oneself twice as quickly as an assistant can for one; and then by having the assistant feed the gold, much time is saved.

For years I used the electric mallet, but have given it up, as it is too slow. I experimented a good deal to perfect it, succeeded in increasing the force of the blow without increasing the magnetic force, increased the polarizing surface of the carbons with increased E. M. F.; introduced a new circular switch, which gave me control of all and each cell, and a new magnetic dynameter, and kept records for months of the E. M. F. and Amps of each cell taken every few days, collecting data for future use, but I was then gradually taking up crystal gold, and found the mallet not suitable, using instead a heavy eight ounce hammer.

You will probably have noticed that burnishing destroys cohesion. Why, I cannot tell you; sometimes it will; at other times it will not. It is this refusal of the pieces to unite that prevents us burnishing in the gold at times; even the experience we have derived from the Herbst process, in 1883, has not given us the required knowledge. In using rotation in condensing gold, sometimes the layers are rendered quite non-cohesive; at other times they are irregularly so, and sometimes quite cohesive. We are told by the inventor that when a layer is found to be non-cohesive, it should be again burnished over by the engine point, and it will become cohesive. I cannot say that I have found it so, although my experience with this process is not very extensive. As it was found that minute portions of gold became attached to the rotating point at first, recourse was had to polishing with fine emery cloth, but latterly the points are made with agate blood-stone, or some equally hard material. I have used aluminium bronze points for the process, but I do not know that it possesses advantages over steel. In the Herbst process, enough gold either large soft cylinders (Wolrab's), or any soft make of gold (personally I prefer crystal), is taken to quite fill the cavity. This can be either burnished in with any point which will enable you to reach all parts of the cavity, or first cover the gold with a piece of cotton wool or Japanese paper, and burnish on that.

The latter process leaves the gold in a more even and better adapted condition, but also leaves it quite non-cohesive.

The cavity can then be scraped with a sharp excavator until cohesion is restored, or a new layer can be attached if desired, by the aid of sharp serrations.

Cohesion may not be necessary if there are still walls to the cavity unfilled, or if you can get sufficient undercut for the new layer. Layer after layer is added until the cavity is quite filled. The process can never be fully accepted, I think, for many reasons: it is not more rapid than self-malleting, less so perhaps than the engine mallet; the process is unmechanical, and has no tendency to inspire the operator with confidence,—in fact, there is an element of uncertainty as to durability that is not encouraging.—*London Dental Record.*

